# Influence of Selective Melanocortin-4 Receptor Antagonist HS014 on Hypersensitivity After Nervous System Injuries in a Model of Rat Neuropathic Pain: A Narrative Review of the Literature

**DOI:** 10.7759/cureus.17681

**Published:** 2021-09-03

**Authors:** Narmeen Shikdar, Faisal Alghamdi

**Affiliations:** 1 General Dentistry, Faculty of Dentistry, King Abdulaziz University, Jeddah, SAU; 2 Oral Biology, Faculty of Dentistry, King Abdulaziz University, Jeddah, SAU

**Keywords:** allodynia, hyperalgesia, hs014, melanocortin 4 receptor, nerve injury, rat neuropathic pain models, review

## Abstract

Background and Objective: The melanocortin-4 (MC4) receptor has been evaluated as a possible new therapeutic for neuropathic pain treatment. The purpose of this review article was to review and evaluate all recent in vivo studies on the effect of the MC4 receptor antagonist HS014 on rat hypersensitivity caused by neuropathic pain.

Methods: An electronic search was carried out using Scopus, Web of Science, PubMed, and Google Scholar. The following inclusion criteria were used: rat models of neuropathic pain-induced hypersensitivity, with investigated effects of the selective antagonist HS014. The included duration of the search was within the last ten years. Data regarding HS014, neuropathic pain model, post-treatment administration time and dose (days post-injury), behavior assessment assays, treatment frequency, and route of delivery were collected and subjected descriptively as complementary data in this narrative review.

Results: This narrative review included four papers that fulfilled the eligibility criteria. The findings demonstrate that as compared to vehicle-treated rats, administration of the MC4 receptor antagonist HS014 remarkably raised paw withdrawal threshold (PWT) in three studies and heat withdrawal latency in four studies among rat models subjected to neuropathic pain.

Conclusions: In rat neuropathic pain models, the MC4 receptor antagonist HS014 is helpful in reducing hypersensitivity. However, further studies are needed to determine the ideal treatment dosage and timing. In addition, further investigations are required for the role of this selective receptor antagonist (HS014) and compared with other types of MC4 receptors in neuropathic pain in humans.

## Introduction and background

Pain is a major public health issue. Pain frequently renders the influenced individual incapable to implement daily activities, and even when not debilitating, the pain has a severe impact on quality of life. Pain in hospitalized patients can lead to longer hospitalizations, longer recovery times, and indigent patient outcomes, all of which have an impact on the quality and cost of health care [1,2]. Positive (allodynia and hyperalgesia) and negative (hypoalgesia and hypoesthesia) symptoms describe neuropathic pain, which is caused by injury to the nerve system. The mechanisms that underpin neuropathic pain are complicated, including both central and peripheral nervous systems [1-3]. To date, there is no successful treatment for neuropathic pain, and the available medication is usually coupled with substantial adverse effects. Furthermore, current neuropathic pain medications have limited effectiveness and typically only give partial pain relief, with only approximately 10% of patients reporting a pain reduction of more than 50% [1].

Pain and nociception have been linked to the melanocortin system [4-8]. Two review studies [9,10] revealed the influences of melanocortin on fever, inflammation, nerve regeneration, and body weight control [11]. Furthermore, the melanocortin system reacts with the opiate system; it can inhibit morphine-induced reduction of evoked potential in frog and cat nerve tissues [12], as well as reduce morphine-induced analgesia [13-16]. Also, antagonists of the melanocortin-4 (MC4) receptor have been found to reduce morphine anti-nociceptive tolerance [17]. Several melanocortins have been demonstrated to have a direct impact on nociception in addition to their interactivity with the opiate system. Hyperalgesia was generated in rats after activation of the MC4 receptor with an endogenous agonist [18-20], while therapy with MC4 receptor antagonists was found to reduce hypersensitivity caused by nerve damage among the rats. Melanocortin receptors are prevalent in mammalian brain areas associated with nociception and pain. The melanocortin-1 receptor (MC1R) and the MC4 receptor have both been implicated in the control of pain [21-23]. The MC1R is mostly described in the periaqueductal gray (PAG) of the brain, where it controls pain [11]. MC4 receptor, on the contrary, is extensively distributed across the nervous system [10,24], along with the dorsal root ganglia [25], PAG [11], and brain [11], and it plays an important role in nociception among different pain rat models [20,24,26,27].

Some studies have shown that HS014 can play a significant role in the development and maintenance of neuropathic pain in rats [28-30]. It was discovered in the past few decades that intraplantar injection of HS014 could weaken nervous system injury-induced allodynia and hyperalgesia, implying that HS014 may play an important role in the formation and transduction of neuropathic pain [25,27]. However, the knowledge of the mechanism by which the MC4 receptor antagonist regulates the process of neuropathic pain remains elusive.

The molecular processes behind the melanocortin system's biological actions in pain remain unknown. Studies into the success of MC4 receptors as a new therapeutic target in the management of neuropathic pain have become possible as the availability of MC4 receptor ligands has increased. There has been no review study comparing the effect of MC4 receptor antagonist HS014 on a rat model of neuropathic pain hypersensitivity. Therefore, the aims of this review article were to: (1) review the effect of MC4 receptor antagonist HS014 on a rat model of neuropathic pain hypersensitivity, with the objective of identifying whether MC4 receptor antagonist HS014 reduces, increases, or has no influence on a rat model of neuropathic pain hypersensitivity; (ii) evaluate whether the effect of MC4 receptor antagonist HS014 on differs between paw withdrawal threshold test (grams; PWT) and paw withdrawal latency test (seconds; PWL) in rats exposed to neuropathic pain compared to vehicle-treated rats. The findings of this study will give evidence for the function of the MC4 receptor as a possible therapeutic target for the successful therapy of neuropathic pain, as well as the rationale for future research into the role of HS014 in neuropathic pain in humans.

## Review

Methods

Literature Search Strategy

An electronic search for articles in the English language was performed using PubMed, Scopus, Web of Science, and Google Scholar from 2011 to 2021 due to the lack of updated and limited published reviews cover this research area. The literature search strategy was carried out in March 2021 and then updated in June 2021. The search for this narrative review was done by using the following electronic databases: Scopus, Web of Science, Public Medline (PubMed), and Google Scholar digital data basis. Communication with the authors of the studies included, for additional data or clarification, was done. The search was conducted using the following combination of keywords and Boolean operators ("AND," "OR"): [(MC4 receptor) OR (melanocortin 4 receptor) OR (HS014) OR (selective antagonist)] AND [(allodynia) OR (hypersensitivity) OR (hyperalgesia) OR (pain)] AND [(rodent) OR (rat)]. 

Eligibility Criteria

Studies were included if they followed the applied criteria: published in vivo animal studies that discuss the effect of MC4R antagonist (HS014) on a rat model of neuropathic pain hypersensitivity; scientific papers published between 2011 and 2021; scientific papers published in the English language; and studies conducted on an extracted rat or rodent animal studies only. On the other hand, studies were excluded if they met any of the following applied criteria: narrative/critical or systematic reviews; in vitro and in situ studies; editorial or personal opinion articles; papers that illustrated HS014 combined with other treatments; papers that illustrated no relevant outcome measures reported; and papers that discussed the role of melanocortin 4 receptor antagonist (HS014) by percentages and samples taken from human or animal (dogs, cats, frogs, rabbits, sheep’s, ferrets, and monkeys) sources.

Data Extraction

The two reviewers independently read the full articles and considered the following variables: title, abstract, material and methods, and main results. The data were then verified for completeness and accuracy and were harvested into a standardized Microsoft Office Excel worksheet. Data were gathered and organized into columns with the following information: study (author and year), sample (number, species, strain, and gender), type of MC4 receptor antagonist, neuropathic pain model, post-treatment administration time and dose (days post-injury), behavior assessment assays, treatment frequency, route of delivery, and important findings. No meta-analysis was applied in this review due to the type of study.

Study Selection

A total of 138 articles were initially obtained through the keywords using the databases. Of those 89 articles were deleted because they displayed either duplicity or unrelated topics and 61 articles were based on abstract and title. Only 49 full-text articles were carefully assessed for eligibility. Out of that, 45 studies were excluded from this extensive review due to the following reasons: narrative/critical or systematic reviews (n=4); in vitro and in situ studies (n=9); editorial or personal opinion articles (n=2); HS014 combined with other treatments (n=11); no relevant outcome measures reported (n=7); and human and animal studies (n=12). Lastly, 4 papers were selected to be included in this review. The literature search strategy for this review has been summarized in Table 1.

**Table 1:** Summary of the literature search strategy

Search strategy		No. of publications
1	Publications retrieved from PubMed database	17
2	Publications retrieved from Scopus database	19
3	Publications retrieved from Web of Science database	24
4	Publications retrieved from Google Scholar	78
5	Total number of publications from electronic search (1+2+3+4)	138
6	Total number of publications after removal of duplicates	89
7	Publications remaining after the title and/or abstract screening	49
8	Publications retrieved through manual search	0
9	Total included publications	4

Results

Study Characteristics

This review included four studies that were randomized animal experiments [20,26,27,30]. All four studies were carried out in China. All of the subjects in their experiments were male rats with sciatic nerve-chronic constriction injury (SCN-CCI) and HS014 as an MC4 receptor antagonist used to treat neuropathic pain [20,26,27,30]. Based on a narrative review of the literature, a discussion of the data is presented to evaluate the influence of HS014 on hypersensitivity in rat models of pain. Because the included studies were conducted on rats, the treatment protocols and study population varied in comparison with those that would be applied to humans. All the investigated rats of neuropathic pain were administrated by HS014, while the vehicle-tread rats (control group) were administrated by saline [20,26,27,30]. The other study characteristics (including strain, gender, type of MC4 receptor antagonist, neuropathic pain model, post-treatment administration time and dose (days post-injury), behavior assessment assays, treatment frequency, route of delivery, and main results) were collected and outlined in Table 2.

**Table 2:** Details of the included studies SCN-CCI: sciatic nerve-chronic constriction injury, MC4: melanocortin-4, PWT: paw withdrawal threshold test, PWL: paw withdrawal latency.

Author/year	Chu et al. [20]	Chu et al. [26]	Chu et al. [27]	Zhao et al. [30]
Number of samples	NR	NR	96	128
Species	Rat	Rat	Rat	Rat
Strain	Wistar	Wistar	Wistar	Sprague-Dawley
Gender	Male	Male	Male	Male
Neuropathic pain model	SCN-CCI	SCN-CCI	SCN-CCI	SCN-CCI
MC4 receptor antagonist	HS014	HS014	HS014	HS014
Time of treatment (days post-injury)	14	Daily 1-7 days	Daily 1-7 days	Daily 3-14 days
Treatment frequency (days)	1	7	7	12
Behavioral testing day(s) post-injury	14	3, 7, 14	3, 7, 14	3, 7, 14
Maximum effect dose, time post-treatment administration	1 µmol, 60 minutes + 1 µmol, 30 minutes	5 µg/day, day 7	5 µg/day, day 7	1 nmol/day, 30 minutes, day 3
Behavior assessment assay	von Frey + thermal (heat)	Thermal (heat)	von Frey + thermal (heat)	von Frey + thermal (heat)
Route of delivery	PAG injection	Intrathecal	Intrathecal	Intrathecal
Main results	The positive effect of (HS014) on PWT and PWL tests in rats exposed to neuropathic pain compared to vehicle-treated rats following nerve injury.	NR regarding the effect of (HS014) on the PWT test, but Positive effect of (HS014) on PWL test in rats exposed to neuropathic pain compared to vehicle-treated rats following nerve injury.	The positive effect of (HS014) on PWT and PWL tests in rats exposed to neuropathic pain compared to vehicle-treated rats following nerve injury.	The positive effect of (HS014) on PWT and PWL tests in rats exposed to neuropathic pain compared to vehicle-treated rats following nerve injury.

Effect of HS014 on PWT After the Nerve Injury in Rats

The von Frey behavioral test's major outcome measure was the PWT (grams). The shortest fiber that caused a behavioral reaction, such as paw withdrawal, is referred to as PWT. Three studies [20,27,30] illustrated that administration of HS014 after the nerve damage has remarkable augmentation in PWT compared to vehicle-treated rats. Only one study was not reported regarding the PWT test [26]. The anti-allodynic influence of HS014 was dose-dependent, and it was spotted at different times post-treatment administrations among the included studies as shown in Table 2.

Effect of HS014 on PWL After the Nerve Injury in Rats

The PWL (seconds) was a major outcome calculated for assessing reactions to thermal (heat or cold) stimuli. PWL is defined as the time it takes for the rat to withdraw the paw after being stimulated with heat (HWL) or cold (CWL). All four studies illustrated that administration of HS014 after the nerve damage has remarkable augmentation in HWL compared to vehicle-treated rats. No study used the CWL as a PWL test in their investigation [20,26,27,30]. The alleviating of the spotted anti-nociceptive effect was dose-dependent, and it was detected at different times post-treatment administrations, depending on the dose applied among the included studies as shown in Table 2.

Discussion

The purpose of this review was to review and evaluate all recent in vivo studies within the last 10 years assessing the effect of HS014 on rat neuropathic pain hypersensitivity. This review included four studies in rats exposed to neuropathic pain compared to vehicle-treated rats between PWT test (grams) and PWL test (seconds).

The results in this narrative review exposed the positive influence of HS014 on mitigating hypersensitivity after the nerve damage in rats. Although only limited articles could be selected in this review, all the articles that investigated the HS014 found that it remarkably reduced heat and mechanical hypersensitivity after the nerve damage in a dose-dependent manner. The findings of four articles [20,26,27,30] illustrated that administration of HS014 remarkably reduced heat and mechanical hypersensitivity after the nerve damage with both the dose and time of assessment post-administration remarkably affecting the anti-nociceptive effects. Furthermore, pre-treatment with HS014 retards the improvement of thermal hyperalgesia and mechanical allodynia after the nerve damage [20]. The findings of three articles [20,27,30] demonstrated that, after the nerve damage, administration of HS014 has remarkable augmentation in PWT. Furthermore, the findings of four studies [20,26,27,30] illustrated that, after the nerve damage, administration of HS014 has remarkable augmentation in HWL.

A study was conducted in 2006; they discovered that HS014 can alleviate mouse hyperalgesia caused by formalin-induced inflammatory pain by reducing nitric oxide overproduction and preventing the cascade response of inflammatory cytokines [31]. Another study found that HS014 can prevent the release of excitatory amino acids (glutamic acid and asparagine acid) by TNF-alpha in activated astrocytes in vitro. Furthermore, it has been illustrated that a toxic increase in excitatory amino acid in vivo can promote N-methyl-D-aspartic acid receptor activation, which can enhance neuron excitability and, ultimately, result in central sensitization [32].

MC4 receptor is found in several central nervous system regions, including astrocytes, brainstem, cortex, hypothalamus, hippocampus, thalamus, and spinal cord. An increasing amount of data suggests that the melanocortin system is involved in synaptic plasticity and nociception [33]. The hypersensitivity induced by melanocortin peptides, influencing pain responses [18] and blocking morphine's analgesic effects [34] was done by melanocortin peptides. Furthermore, in a rat model of neuropathic pain, spinal melanocortin receptors were shown to be increased and inhibiting the MC4 receptor resulted in anti-allodynia, indicating that the melanocortin system is implicated in neuropathic pain.

In our review, we found that the period of evaluation following administration had a significant influence on the antagonist's anti-nociceptive efficacy. After the nerve injury, pre-treatment with HS014 slowed the improvement of thermal hyperalgesia and mechanical allodynia [20]. These findings imply that HS014 might be a promising therapeutic target for neuropathic pain treatment [20,26,27,30]. It is crucial to note, however, that these findings are depended on a small number of articles. As a result, further study using standardized protocols is required to determine the best beneficial dose and timing of administration. Furthermore, there is a direct interpretation of these findings into clinical practice may be impossible. The dosages that elicit analgesic effects in animal models may not yield the same results in humans. Furthermore, prior to clinical trials, other and less intrusive techniques of administration must be studied. The findings of our narrative review contribute significantly to our understanding of the influence of HS014 on hypersensitivity after the nerve injury in rats exposed to neuropathic pain compared to vehicle-treated rats; however, more study is required before these findings may be applied in clinical trials.

There are some limitations resulting from the narrative nature of the review: although searching the most major databases in the area of health sciences, we may not cover all published papers. However, we think that this risk has been reduced as a result of the restricted search strategy applied, the manual search of references and the double independent review procedure applied. Animal studies are frequently spread in nature, and as a result, some of the factors associating with the spotted differences such as time and dose of HS014 tested after the nerve injury in the synthesized investigations. Furthermore, the small number of articles selected (n=4) and restricted to a particular MC4 receptor antagonist (HS014) may have influenced the results, indicating that further research is needed in this field.

## Conclusions

It can be concluded that the MC4R antagonist HS014 significantly reduced thermal and mechanical hypersensitivity caused by nerve damage in a dose-dependent manner. Furthermore, pre-treatment with HS014 slowed the onset of thermal hyperalgesia and mechanical allodynia after nerve damage. However, the ideal therapy dosage and timing are unclear. Therefore, further investigations are required for the role of this selective receptor antagonist (HS014) and compared with other types of MC4 receptors in neuropathic pain in humans.
